# A genome-wide association study for harness racing success in the Norwegian-Swedish coldblooded trotter reveals genes for learning and energy metabolism

**DOI:** 10.1186/s12863-018-0670-3

**Published:** 2018-08-29

**Authors:** Brandon D. Velie, Kim Jäderkvist Fegraeus, Marina Solé, Maria K. Rosengren, Knut H. Røed, Carl-Fredrik Ihler, Eric Strand, Gabriella Lindgren

**Affiliations:** 1 Department of Animal Breeding & Genetics, Swedish University of Agricultural Sciences, Uppsala, Sweden; 2 Department of Basic Sciences and Aquatic Medicine, Norwegian University of Life Sciences, Oslo, Norway; 3 Department of Companion Animal Clinical Sciences, Norwegian School of Veterinary Science, Oslo, Norway; 4 Department of Biosystems, KU Leuven, 3001 Leuven, Belgium

**Keywords:** Genetic, Horse, Performance, Racehorse

## Abstract

**Background:**

Although harness racing is of high economic importance to the global equine industry, significant genomic resources have yet to be applied to mapping harness racing success. To identify genomic regions associated with harness racing success, the current study performs genome-wide association analyses with three racing performance traits in the Norwegian-Swedish Coldblooded Trotter using the 670 K Axiom Equine Genotyping Array.

**Results:**

Following quality control, 613 horses and 359,635 SNPs were retained for further analysis. After strict Bonferroni correction, nine genome-wide significant SNPs were identified for career earnings. No genome-wide significant SNPs were identified for number of gallops or best km time. However, four suggestive genome-wide significant SNPs were identified for number of gallops, while 19 were identified for best km time. Multiple genes related to intelligence, energy metabolism, and immune function were identified as potential candidate genes for harness racing success.

**Conclusions:**

Apart from the physiological requirements needed for a harness racing horse to be successful, the results of the current study also advocate learning ability and memory as important elements for harness racing success. Further exploration into the mental capacity required for a horse to achieve racing success is likely warranted.

**Electronic supplementary material:**

The online version of this article (10.1186/s12863-018-0670-3) contains supplementary material, which is available to authorized users.

## Background

Regardless of horseracing discipline, speed, or perhaps more appropriately, unparalleled speed, is the “holy grail” of almost every horse owner, trainer, and breeder. However, speed alone does not necessarily equate to success on the racecourse. The manner in which a horse demonstrates speed is critical to its racing success [1–3]. For example, while the ability to gallop fast may result in a champion Thoroughbred (TB) or Quarter Horse (QH), the same ability in a Standardbred (SB) or Coldblooded Trotter (CT) is of little value. In SB and CT racing, horses undoubtedly require speed, but galloping, a four-beat gait, results in disqualification [1–3]. Thus, speed in these breeds must be demonstrated at trot, a contralateral two-beat gait, or pace, an ipsilateral two-beat gait [1–4]. Consequently, racing success in SBs, CTs, and other harness racing breeds depends not on an individual’s capacity for speed, but on an individual’s capacity for speed in a specific gait.

To date, significant genomic resources have been applied in studies attempting to map speed and racing success in TBs and QHs [4–18]. While these studies have proven to be of great value for gallop racing breeds, their applicability to harness racing breeds has been limited [19–21]. In fact, a genomic study exploring locomotion pattern in Icelandic horses is arguably the most influential study to date when it comes to racing success in harness racing breeds [22]. The study discovered that a premature stop codon in the doublesex and mab-3 related transcription factor 3 (*DMRT3*) gene had a major effect on the pattern of locomotion in the horse, resulting in some horses possessing the ability to trot or pace at high speed without transitioning into gallop [22]. Follow-up studies were then able to demonstrate that for many harness racing breeds the *DMRT3* mutation was associated with racing success (e.g. increased earned prize money) [20, 23–26]. However, the mutation appears to only account for between 0 and 6.3% of the phenotypic variation in traits widely used to evaluate racing success [24, 26]. Considering that heritability estimates for some of these traits are as high as 0.38, the likelihood of other genes playing a significant role in a harness racing horse’s success is high [20, 23, 27–38]. Nevertheless, genome-wide association (GWA) studies with harness racing performance are lacking [23].

Despite the fact that harness racing success is of high economic importance to the global equine industry, genome scans for performance appear to predominantly target Thoroughbreds and sport horses (e.g. Warmbloods) [4–6, 8–14, 39–41]. Most studies involving harness racing breeds tend to focus on detecting genes underlying certain conditions and diseases [42–47]. While disease studies are undoubtedly important for improved animal well-being, a greater awareness of the genes, and by extension the underlying biological mechanisms, involved in racing success are also likely to prove highly valuable. A deeper understanding of the biology underpinning success in a racehorse ultimately can lead to more targeted medical treatments as well as a heightened awareness of the physical limitations (e.g. lack of speed at trot) some horses will inevitably possess.

Motivated by these facts, we conducted a GWA study to identify genomic regions and genes associated with harness racing success using the Norwegian-Swedish Coldblooded Trotter (NSCT). Norwegian-Swedish Coldblooded Trotters are ideally suited for GWA analyses of harness racing performance as their small population is not only likely to correspond with low within breed genetic variation, but the limited region in which NSCTs are eligible to race is also likely to reduce environmental variation. Thus, a more accurate assessment of the relationship between genomic regions and harness racing performance is achievable.

## Methods

### Data collection

Pedigree information and performance data on all raced and unraced NSCTs born between 1 January 2000 and 31 December 2009 were provided by the trotting associations in both Norway and Sweden (Norsk Rikstoto and Svensk Travsport). Pedigree information included horse name, horse id, date of birth, country of birth, sex, breeder, sire id, and dam id. Performance data, as of 8 February 2017, was presented per race per horse (i.e. data included individual race records with each record corresponding to a given horse’s specific performance in the race). This data included non-competitive premie and qualification races, with each record containing information on horse id, race date, race track, race type, race distance, trainer, owner, driver, finish position, prize money earned, gallop status, and average km time [48, 49].

### Sample acquisition

In order to reflect the raced population as accurately as possible, a list of raced individuals was randomly generated from the data described above using the statistical software R [50]. In addition to having raced, two requirements were set for inclusion in the study: 1. Hair and/or blood samples had to be readily accessible from the pedigree registration authorities in either Norway (Department of Basic Sciences and Aquatic Medicine, Norwegian University of Life Sciences) or Sweden (Animal Genetics Laboratory, Swedish University of Agricultural Sciences) 2. Sufficient sample material had to be available to ensure DNA quality standards would likely be achieved. The first 661 horses on the list that met these criteria were included in the study. Average relatedness within the selected group, estimated using the genetic software Contribution, Inbreeding, Coancestry, was 0.16 (interquartile range [IQR] 0.11–0.18) [51].

### DNA isolation

Deoxyribonucleic acid was prepared from the hair roots using a standard hair-preparation procedure. Briefly, 186 μL Chelex 100 Resin (Bio-Rad Laboratories, Hercules, CA) and 14 μL of proteinase K (20 mg/mL; Merck KgaA, Darmstadt, Germany) were added to the sample. The mix was incubated at 56 °C for 2 h and the proteinase K was inactivated for 10 min at 95 °C. For DNA preparation from blood samples, 350 μL of blood was used and isolated on the Qiasymphony instrument using the Qiasymphony DSP DNA mini kit (Qiagen, Hilden, Germany). A summary of the final horses selected for genotyping is shown in Table 1.

**Table 1:** Summary information for the genotyped and the final sample of horses

	Genotyped Horses	Final GWA Horses
	N	N
Sex		
Intact males	70	62
Females	271	247
Geldings	320	304
Country of birth		
Norway	360	312
Sweden	301	301
Year of birth		
2000	45	36
2001	73	66
2002	84	80
2003	69	64
2004	56	49
2005	62	55
2006	70	67
2007	71	70
2008	63	59
2009	68	67
Total	661	613

*GWA* Genome-wide association

### Genotyping and quality control

Prior to quality control (QC) the data set consisted of individuals genotyped using the 670 K Axiom Equine Genotyping Array (*n* = 570) and the 670 K+ Axiom Equine Genotyping Array (*n* = 91). The data from the two arrays were subsequently merged based on SNP name, chromosome, and position, yielding a combined SNP data set of 611,888 SNPs for 661 horses. Iterative QC was then performed with the GenABEL package in R to remove poorly genotyped and noisy data using the following thresholds: minor allele frequency (MAF) (< 0.5%), missing genotypes per single nucleotide polymorphism (SNP) (> 5%), missing SNPs per sample (> 15%), and Hardy-Weinberg equilibrium (HWE) (first QC *p* < 1e^− 10^; second QC FDR < 0.2) [50].

### Phenotyping

Pedigree and performance data for the 661 genotyped horses were structured for analyses using custom scripts written in Perl v5.20.1. Sex classifications were based on the official sex of the horse at the end of the study. Number of career starts was defined as the total number of competitive races for each individual (i.e. premie and qualification races were excluded). Number of gallops (NG) was defined as the total number of competitive races in which the horse was recorded as galloping at some point during the race. Career earnings (CE) were calculated as the total amount of prize money won for each horse as of 8 February 2017. Prize money won in Sweden (SEK) was converted to Norwegian currency (NOK) based on the average exchange rate for the year in which the race occurred [52]. Horses having participated in only premie or qualification races were given a value for career earnings of − 1 NOK in order to distinguish them from horses that had zero career earnings, despite having raced competitively. Best km time (BT), independent of starting method, was defined as the fastest average km time for a competitive race in which the horse was not recorded as galloping during the race (i.e. races in which a horse galloped, regardless of if the horse was disqualified, were excluded).

### Genome-wide association analyses

**Fig. 1:**
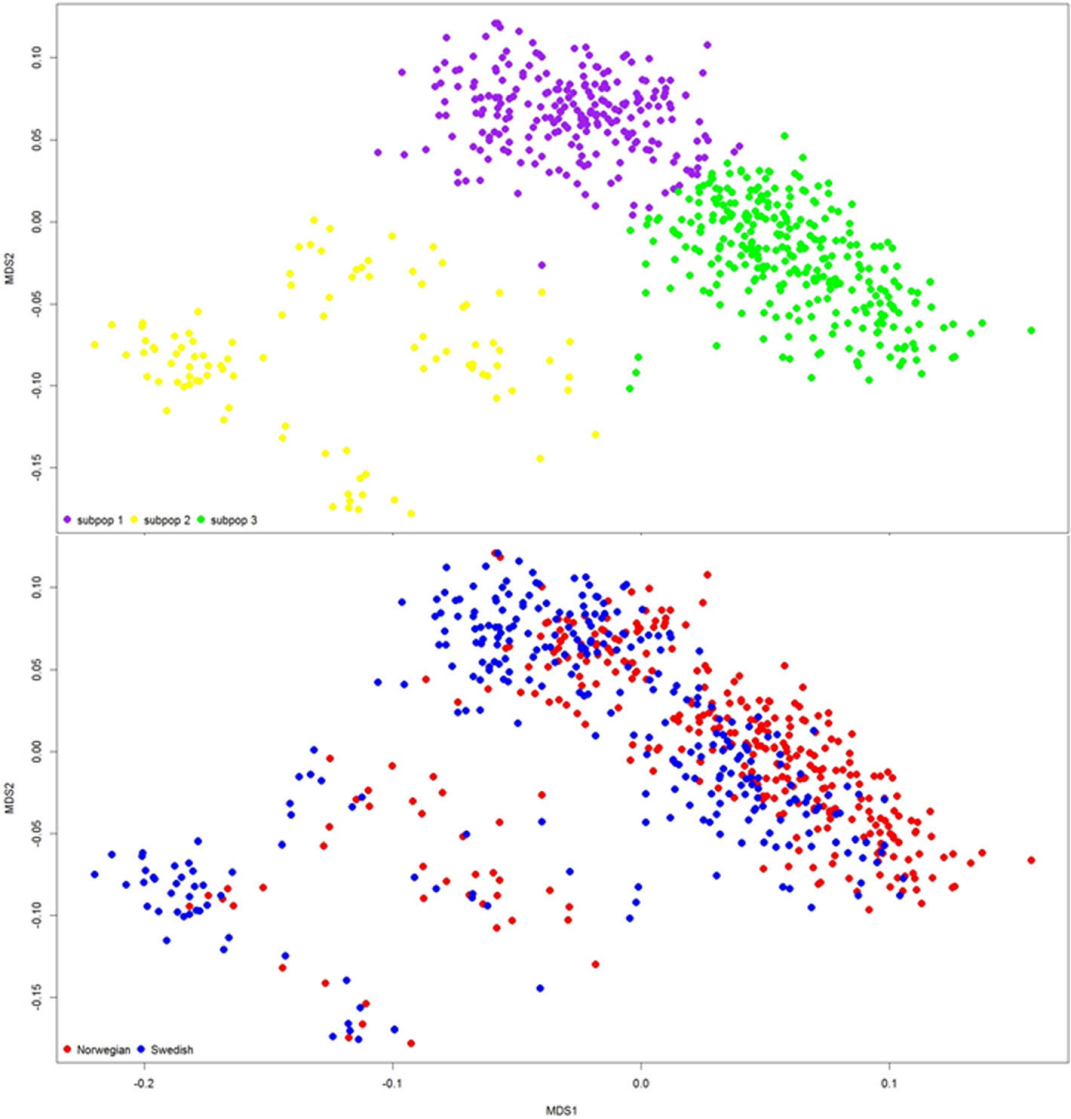
Visualization of multidimensional scaling, stratified by country of birth and subpopulation, performed on the genomic-kinship matrix

Genome-wide association (GWA) analyses were performed using the GenABEL package in R [50]. The package was used to compute an autosomal genomic kinship matrix as well as perform standard K-means clustering. K-means clustering with K = {1,2,…,10} were executed to determine the number of clusters (subpopulations). For each iteration, the sum of within-cluster sum of squares (∑WCSS) was calculated and subsequently plotted vs. K. To define the subpopulations, the number of clusters corresponding with the first inflection point (K = 3) was chosen [53]. The multidimensional scaling (MDS) plot yielded no apparent outliers and a visualization of the genomic-kinship matrix and subpopulations using MDS can be seen in Fig. 1.

To account for any population stratification, genome-wide association analyses of CE, BT, and NG were performed using a mixed model-structured association approach (“mmscore” function with the “strata” option in GenABEL). Preliminary analyses indicated significant effects of sex, birth year, and number of career starts on all three traits. Country of birth was also shown to influence CE. As a result, sex, birth year, number of career starts, and country of birth were included as co-variants in the final analyses accordingly. Genome-wide significance for each of the analyses was determined by Bonferroni correction (*p* < 1.39 × 10^− 7^, corrected for total number of SNPs post QC) while a “suggestive” genome-wide significance threshold was also set at 1.0 × 10^− 5^. Manhattan and quantile-quantile plots were generated in R while the extent of linkage disequilibrium (LD) was estimated by calculating r^2^ values between all pairs of SNPs with inter-SNP distances of less than 1 Mb using PLINK v1.09 (http://zzz.bwh.harvard.edu/plink/). The effB in the GenABEL result was regarded as the allele substitution effect and the proportion of phenotypic variance explained by each significant SNP was estimated as follows:

$$ \mathrm{VAR}\left(\%\right)=\frac{2 pq{\beta}^2}{S^2}\ast 100 $$

Where *p* and *q* are the allele frequencies, *β* is the estimated allele substitution effect, and *S*^2^ is the sample phenotypic variance. Stepwise regressions were then performed to estimate the total proportion of phenotypic variation explained by the multiple genome-wide associated SNPs (before and after pruning at r^2^ > 0.2; PLINK command –indep-pairwaise 100 25 0.2) for each trait using the lm function in R [50]. The bioinformatics database Ensembl (http://www.ensembl.org/) was used for candidate gene screening. Genomic coordinates of genome-wide significant and suggestive genome-wide signficant SNPs +/− 500 kb were used as inputs to generate a list of annotated genes using the Ensembl Biomart function. The PANTHER Classification system was then used to obtain an overview of the biological processes, molecular functions, and pathways known to be affected by these genes [54, 55].

## Results

**Fig. 2:**
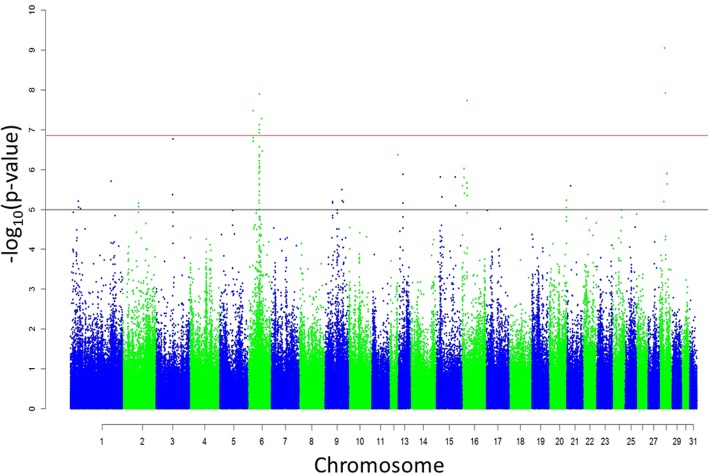
Manhattan plot of the genome-wide association analysis of career earnings. The red horizontal line indicates the genome-wide significance level and the black horizontal line indicates the suggestive genome-wide significance level. Uncorrected λ = 1.0532

**Fig. 3:**
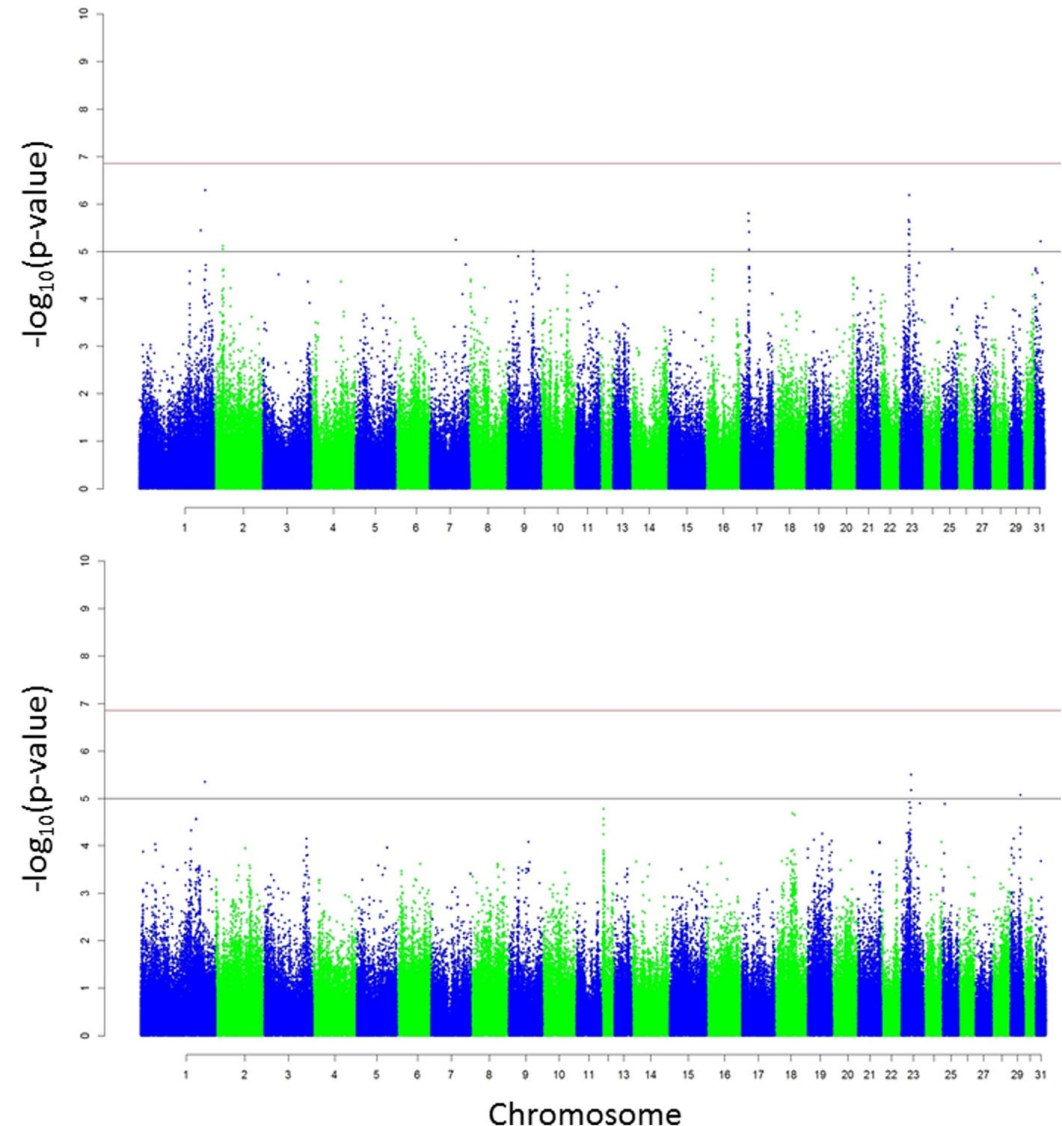
Manhattan plots of the genome-wide association analyses of best km time and number of gallops. The red horizontal lines indicate the genome-wide significance levels and the black horizontal lines indicate the suggestive genome-wide significance levels. Top panel: Best km time analysis, uncorrected λ = 1.0902. Bottom panel: Number of gallops analysis, uncorrected λ = 1.0256

**Fig. 4:**
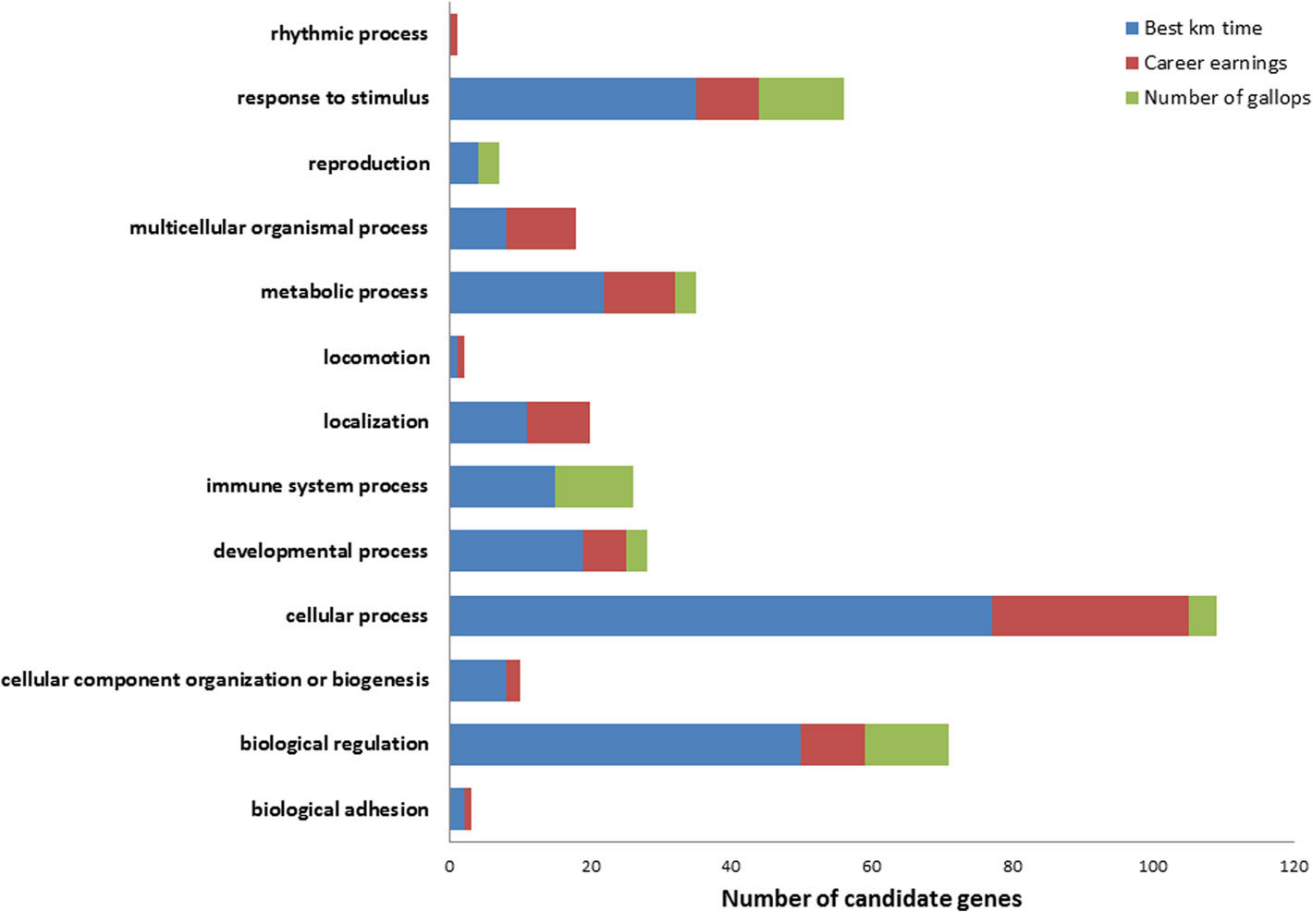
Biological process summary information from the functional classification analysis of candidate genes in PANTHER. PANTHER biological process classification: the function of the protein in the context of a larger network of proteins that interact to accomplish a process at the level of the cell or organism

**Fig. 5:**
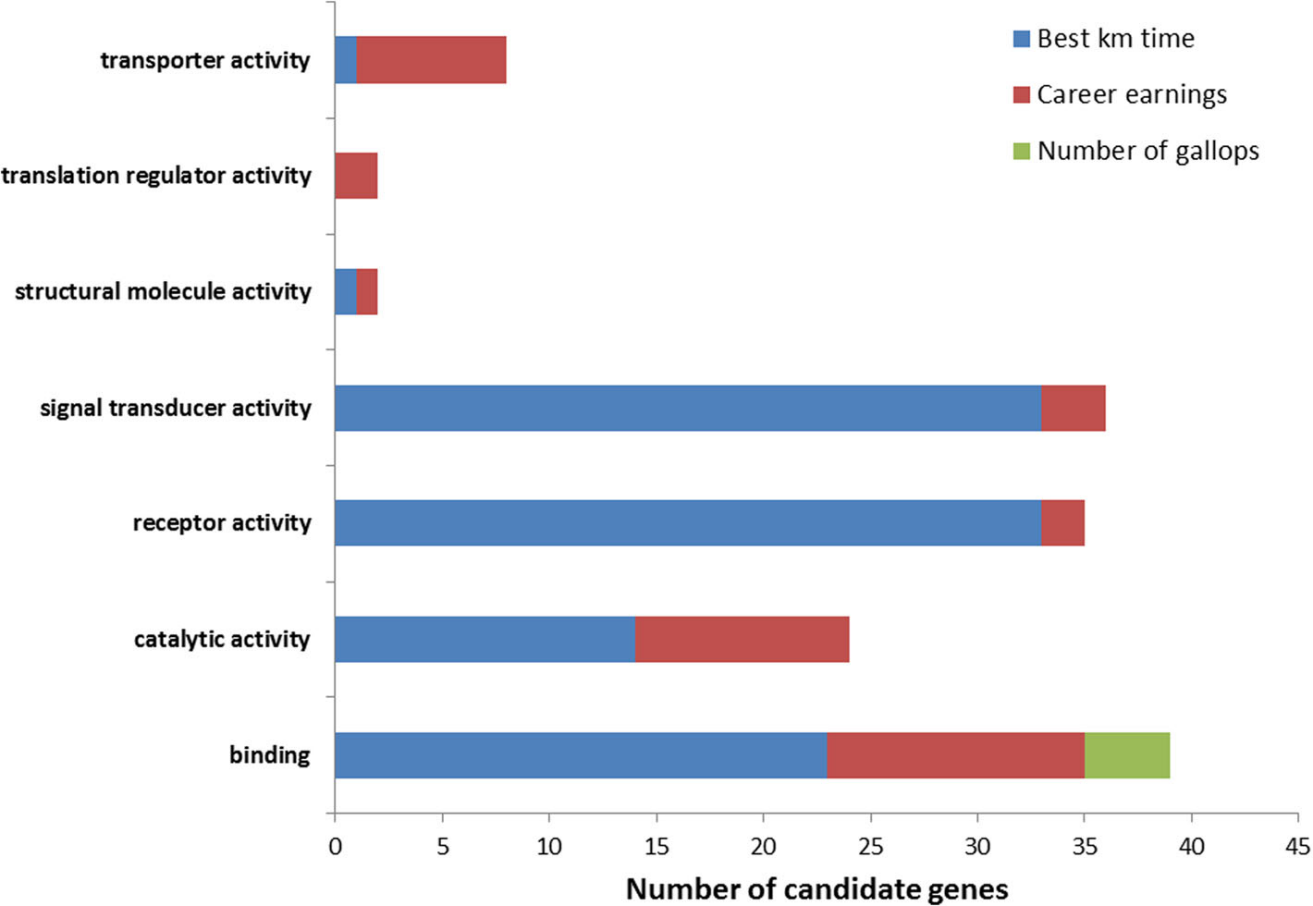
Molecular function summary information from the functional classification analysis of candidate genes in PANTHER. PANTHER molecular function classification: the function of the protein by itself or with directly interacting proteins at a biochemical level

**Fig. 6:**
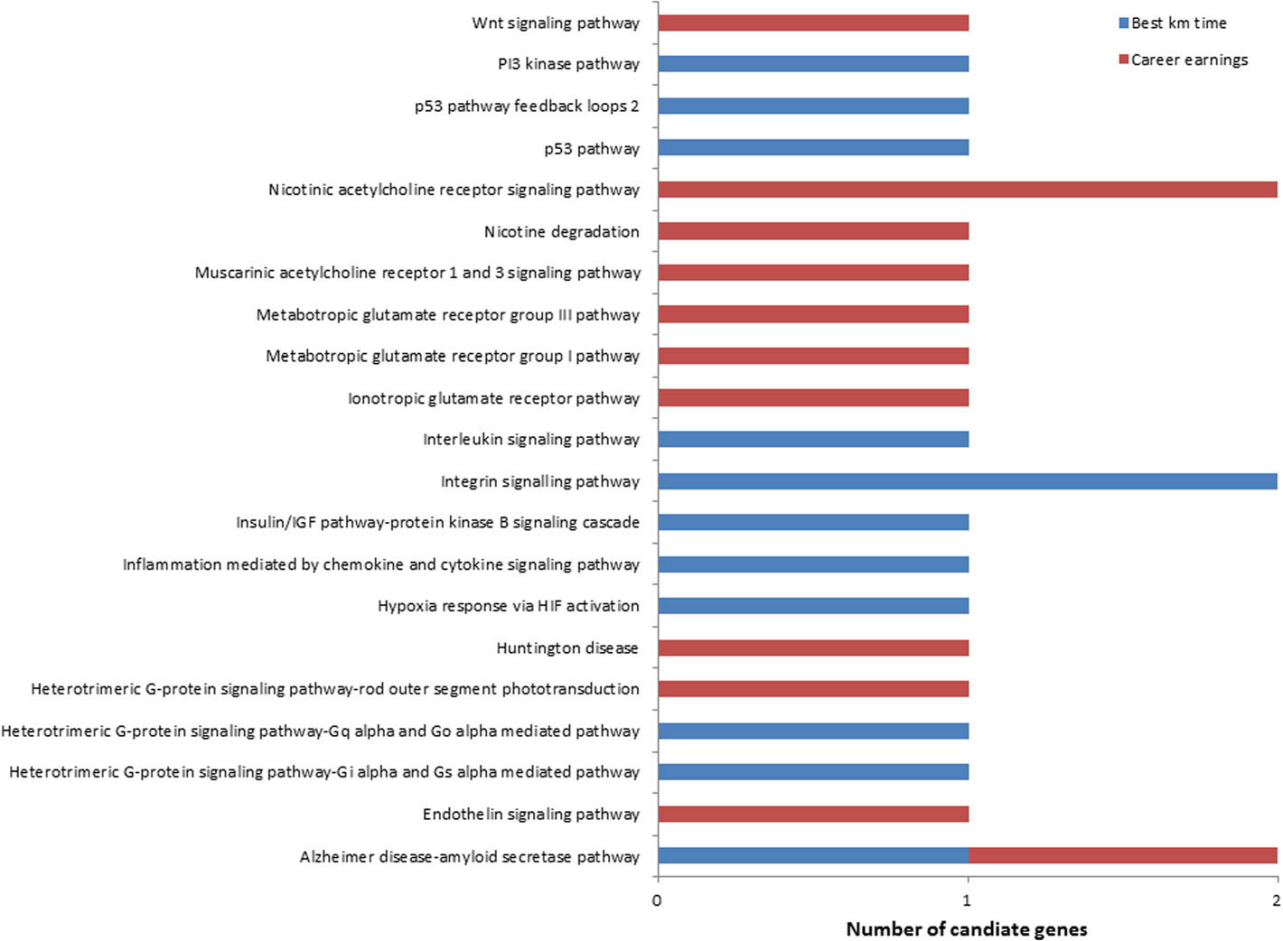
Pathway summary information from the functional classification analysis of candidate genes in PANTHER

Following QC, 359,635 autosomal SNPs and 642 horses were available for association analyses. Of these individuals, 29 had only participated in non-competitive premie and/or qualification races. Initial association analyses with CE, combined with the vast array of reasons known to prevent a horse from competitive racing suggested the exclusion of these horses would help to reduce noise in the final analyses. As a result, only 613 horses, representing 120 sires (interquartile range [IQR] 1–5) and 547 dams (IQR 1–1), were included in the final GWA analyses (Table 1). Descriptive statistics for CE, BT, and NG in the final sample are presented in Table 2. Both CE and NG were not normally distributed and were subsequently log transformed for the GWA analyses. The extent of LD decayed faster across the final sample of horses with mean r^2^ dropping below 0.20 by 3 kb (Additional file 1). The GWA analysis of CE yielded multiple genome-wide significant SNPs (*p* < 1.39 × 10^− 7^), with the majority of these SNPs residing on *Equus caballus* chromosome (ECA) 6 (Fig. 2; Table 3; Additional file 2). Analyses of both BT and NG failed to result in genome-wide significant SNPs. However, two regions of interest were apparent based on the presence of slight peaks on ECA17 and ECA23 in the resulting Manhattan plots (Fig. 3; Additional file 2). Genome-wide significant SNPs for CE, suggestive genome-wide significant SNPs for BT and NG, and the nearest genes are shown in Table 3. The most significant SNP was detected at the 20,006,740 position on chromosome 28 (AX-104828170, *p* = 9.01E-10) and presented an estimated allele substitution effect of − 7079.46 NOK. The favourable allele appeared to be the T allele, with each C allele resulting in a negative effect on CE. Despite the fact that the frequency of the T allele was 98.2% and the frequency of the C allele was only 1.8%, the percentage of phenotypic variance explained by this SNP was 3.85%. The 32 SNPs in total were estimated to explain 18.34%, 18.71% and 33.17% of the variation for CE, NG and BT, respectively, in the population studied. Consequently, after LD pruning, only 17 SNPs remained – explaining 14.17%, 18.38% and 33.13% of the variation for CE, NG and BT, respectively (Table 3). Overall, 378, 144, and 23 candidate genes identified were associated with known biological processes, molecular functions, and pathways, respectively (Figs. 4, 5 and 6; Additional file 3).

**Table 2:** Descriptive statistics of CE, BT, NG for the final sample of horses (*n* = 613)

	Min	25th percentile	Median	Mean	75th percentile	Max
Career earnings (NOK)	0	36,321	124,625	302,506	327,493	4,216,554
Best km time (s)	78.9	86.8	89.3	89.6	92.4	105.7
Number of gallops	0	5	11	14.4	21	123

*CE* career earnings, *BT* Best km time, *NG* number of gallops

**Table 3:** Genome-wide significant SNPs for CE and suggestive genome-wide significant SNPs for BT and NG

SNP	Analysis	Location (ECA:bp)	MAF	*β*	SE	Var (%)	Nearest gene	Distance to nearest gene (bp)	Raw *p*-value
AX-104828170	CE	28:20,006,740	0.018	−3.380	0.552	3.85	*NDUFA12*	114,732	9.01E-10
AX-103147507	CE	28:22,153,465	0.005	−5.762	1.011	3.27	*ENSECAG00000025907*	46,196	1.20E-08
AX-104611735	CE	6:47,132,529	0.015	−3.407	0.599	3.16	*PDE3A*	0	1.28E-08
AX-104865129	CE	16:18,366,321	0.008	−4.488	0.798	3.17	*ENSECAG00000003087/PROK2*	138,149/138,791	1.87E-08
AX-104494389	CE	6:20,020,914	0.006	−4.557	0.825	3.32	*INPP5D*	0	3.36E-08
AX-103248294	CE	6:49,512,490	0.016	−3.166	0.582	3.11	*SOX5*	0	5.28E-08
AX-103090138	CE	6:42,063,985	0.018	−3.007	0.559	2.46	*PLBD1*	3191	7.48E-08
AX-104711589	CE	6:41,462,481	0.012	−3.497	0.657	2.59	*GRIN2B*	0	9.99E-08
AX-104307051	CE	6:41,329,519	0.012	−3.474	0.657	2.67	*GRIN2B*	0	1.21E-07
AX-104144838	NG	23:23,333,501	0.278	−0.214	0.046	1.51	*ENSECAG00000023609*	187,643	3.18E-06
AX-104568609	NG	1:159,285,045	0.004	−1.343	0.293	1.14	*ENSECAG00000003696/ENSECAG00000022264*	19,813/25,804	4.52E-06
AX-102982528	NG	23:23,324,996	0.412	−0.186	0.041	1.27	*ENSECAG00000023609*	179,138	6.74E-06
AX-103734745	NG	29:24,530,437	0.453	−0.176	0.040	1.29	*ENSECAG00000004576*	944,126	8.67E-06
AX-104373992	BT	1:162,993,722	0.016	3.289	0.655	1.79	*ENSECAG00000023062/ENSECAG00000008721*	32,728/51,254	5.10E-07
AX-103261370	BT	23:22,522,071	0.409	−0.910	0.183	0.87	*DOCK8*	0	6.49E-07
AX-104219924	BT	17:19,525,955	0.279	0.946	0.197	2.24	*WDFY2*	0	1.57E-06
AX-104634248	BT	23:21,857,316	0.108	1.346	0.284	1.21	*PIP5K1B/FAM122A*	14,893/18,794	2.20E-06
AX-103287280	BT	23:21,064,571	0.117	1.270	0.268	1.49	*PTAR1*	43,300	2.22E-06
AX-104645782	BT	17:19,318,167	0.258	0.951	0.201	2.21	*ATP7B*	0	2.32E-06
AX-103762427	BT	23:22,461,979	0.154	−1.156	0.245	1.37	*DOCK8*	0	2.45E-06
AX-103530176	BT	23:22,464,604	0.156	−1.139	0.246	1.25	*DOCK8*	0	3.49E-06
AX-103445942	BT	1:151,919,692	0.012	3.600	0.777	1.39	*ENSECAG00000012236/ENSECAG00000013533*	74,605/78,356	3.63E-06
AX-104538418	BT	17:21,083,126	0.279	0.905	0.196	2.29	*KCNRG*	0	3.99E-06
AX-104268231	BT	23:21,689,609	0.385	−0.823	0.179	0.79	*PIP5K1B*	0	4.25E-06
AX-102964033	BT	23:22,423,197	0.121	−1.259	0.275	0.95	*DOCK8*	5699	4.59E-06
AX-104117851	BT	7:65,266,179	0.010	3.724	0.821	1.76	*ENSECAG00000007398*	162,661	5.78E-06
AX-104642194	BT	31:14,300,483	0.006	4.612	1.021	1.37	*MTRF1L/FBXO5*	108/14,491	6.31E-06
AX-103166989	BT	23:22,496,787	0.116	−1.270	0.283	0.99	*DOCK8*	0	7.07E-06
AX-103803214	BT	2:21,466,714	0.049	1.725	0.386	1.82	*AGO1*	2494	7.72E-06
AX-104450418	BT	2:21,311,680	0.087	1.351	0.304	1.93	*TEKT2/ADPRHL2*	10,587/14,440	9.00E-06
AX-103305676	BT	25:26,866,219	0.019	2.590	0.584	1.67	*OR1L3*	492	9.11E-06
AX-104591507	BT	17:20,813,164	0.249	0.898	0.203	1.84	*KCNRG*	227,34	9.36E-06

*CE* career earnings, *BT* Best km time, *NG* number of gallop, *MAF* Minor allele frequency, *β* Estimated allele substitution effect, *Var (%)* Percentage of phenotypic variance explained

Red line = Bonferroni threshold (*P* < 1.39 × 10^−7^)

## Discussion

Knowing where, why, and how genes and athletic prowess intersect in a racehorse has long been the goal of countless researchers, veterinarians, breeders, trainers, and owners [4–38]. While great strides in this area have recently been made for gallop racing horses, similar advancements for harness racing horses have been limited [4–26]. Using the NSCT, the current study explored the genetic background for athletic prowess in a harness racing horse by performing GWA analyses and functional classification for three traits associated with harness racing success. These analyses resulted in a total of 32 SNPs of interest with 9 demonstrating genome-wide significance and 13 residing in genes. Subsequent functional classifications went on to provide further support of the complexity of harness racing success with several candidate genes involved in neurological, metabolic, and musculoskeletal regulation identified. Since a gene can be declared as a candidate gene if at least 1 out of the 4 following characteristics are present: 1) the gene has a known physiological role in the phenotype of interest, 2) the gene affects the trait in question based on studies of knockouts, mutations, or transgenics in other species, 3) The gene is preferentially expressed in organs related to the quantitative trait, or 4) the gene is preferentially expressed during developmental stages related to the phenotype, a large fraction of the genes identified in the current study can plausibly be considered as candidate genes [56]. As a result, the following discussion prioritizes genes that contained variants with significant or suggestive associations with our traits of interest. The rationale for this prioritization is simply that for associated variants that reside outside of annotated genes, it is in general more difficult to determine which gene(s) the variants act on.

### Glutamate ionotropic receptor NMDA type subunit 2B (*GRIN2B*)

Two genome-wide significant SNPs associated with career earnings were located in the *GRIN2B* gene, a gene also identified in a previous study exploring pacing ability in Icelandic horses [57]. The gene has been shown to be involved in neural regulations in humans and laboratory species with mutations in the gene having been associated with neurodevelopmental disorders [58–60]. Considered to be an important factor for learning and memory, one can only speculate as to its association with career earnings in a harness racing horse. However, horses with a greater capacity to learn and adapt to the highly variable nuances of harness racing would conceivably be more likely to achieve racing success. On the other hand, the gene’s association with attention-deficit/hyperactivity disorder in humans suggests that perhaps certain horses lack the ability to focus on racing and training, thereby preventing or at least hindering their racing performance [61].

### ATPase copper transporting beta (*ATP7B*)

A single suggestive genome-wide significant SNP associated with best time was located in the *ATP7B* gene on ECA17. The gene encodes a protein that functions as a monomer, exporting copper out of cells. Excess copper can cause serious toxicity with the process of excess copper disposal relying heavily on *ATP7B* [62, 63]. Over 500 mutations have been identified in the gene, 380 of which are considered to be disease causing mutations [64]. Elevated levels of copper in the body often result in muscle stiffness with acute muscle stiffness prior to a race having the potential to affect individual performance and chronic muscle stiffness likely to impact a horse’s conditioning, trainability, and overall capacity for speed [62, 63].

### Potassium channel regulator (*KCNRG*)

The *KCNRG* gene on ECA17 was also identified by two SNPs demonstrating suggestive genome-wide significance for best time. The gene encodes a protein which regulates the activity of voltage-gated potassium channels with a study using songbirds suggesting potassium channels to be lineage specific [64, 65]. The same study also revealed that apart from broad expression in the brain a subset of potassium channel genes are selectively expressed, with the authors hypothesizing that the *KCNRG* gene may be associated with learning [65]. Although no previous studies of racehorse performance have specifically identified *KCNRG* as important for racing success, a large conserved haplotype on ECA17 has been advocated to have selective importance in Thoroughbreds and closely related breeds [4, 66].

Also of note is the role voltage-gate potassium channels play in Hyperkalemic periodic paralysis (HYPP), a genetic disorder predominantly seen in Quarter Horses. The condition, caused by a mutation in the sodium voltage-gated channel alpha subunit 4 (*SCN4A*) gene, manifests intermittently with clinical signs ranging from muscle fasciculation to signs of paresis [67–70]. Hyperkalemia, a term used to describe abnormally high levels of potassium in the blood, is often seen during or immediately after an attack. Voltage-gated potassium channels are thought to remain open, allowing continual potassium efflux, thereby promoting an open sodium channel configuration. As a result, an HYPP attack can be triggered or an already occurring attack can increase in severity [67, 70–72]. Horses with HYPP also tend to possess hypertrophic muscles; however, they have been reported as having a reduced tolerance to exercise with relatively more lactate being produced during exercise [73, 74].

### Phosphatidylinositol-4-phosphate 5-kinase type 1 beta (*PIP5K1B*)

A single suggestive genome-wide significant SNP associated with best time was also located in the *PIP5K1B* gene on ECA23. Three widely expressed isoforms of *PIP5K1* are responsible for the regulation of the major pools of cellular phosphatidylinostitols in mammalian tissues, with *PIP5K1B* negatively regulated in response to oxidative stress [75, 76]. Neurite outgrowth, a critical process for neuronal development, has also been shown as negatively regulated by *PIP5K1A* [77]. Since the current study is the first to suggest an association between *PIP5K1B* and racing success, understanding the roles *PIP5K1* isoforms have on a horse’s capacity for speed remains a task for future studies. However, genes that influence cell differentiation processes, such as endocytosis, assuredly contribute in some way or another to the physical limitations and overall performance of any racehorse.

### Dedicator of cytokinesis 8 (*DOCK8*)

Perhaps the most obvious candidate gene for harness racing success in the current study was *DOCK8*. Five suggestive genome-wide significant SNPs indicated the importance of *DOCK8* to a horse’s best time, with 4 of the SNPs located in the gene. Mutations in *DOCK8* result in a form of hyper-IgE syndrome; however, loss or mutations of *DOCK8* have also been associated with intelligence and motor retardation [78–83]. While the importance of intelligence in a racehorse has been briefly discussed above, in the case of *DOCK8* the significance of the gene may lie with its link to motor skills. *DOCK8* is not only located on ECA23, the same chromosome as *DMRT3*, but multiple studies have hypothesized some sort of commonality or overlap between *DMRT*-(1,2,3) gene effects and *DOCK8* [22, 82, 84]. Despite the established association between *DMRT3* and harness racing performance, additional research of *DMRT3* in horses strongly suggest that the mutation is unlikely to be the single cause of gaiting ability [22, 53, 85–88]. Therefore, it is conceivable that *DOCK8* also significantly contributes to gaiting ability, ultimately playing some role in a harness racing horse’s propensity to exhibit speed at trot or pace.

### Phosphodiesterase 3A (*PDE3A*)

The protein encoded by the *PDE3A* gene, a gene on ECA6 in which a single genome-wide significant SNP associated with career earnings is located, plays a critical role in cardiovascular function [89–91]. The encoded protein regulates vascular smooth muscle contraction and relaxation and has been linked to familial hypertension, cardiovascular disease, and fertility [89–92]. Healthy cardiovascular function is important for racing success as the act of racing undeniably requires a higher than resting-level of oxygen to support the horse’s increased muscle activity. A mutation in the *PDE3A* gene that ultimately alters cardiovascular function could potentially prevent a horse from meeting the higher metabolic demands of racing, thus decreasing his/her chances of winning and limiting his/her career earnings. On the contrary, an advantageous mutation in the gene could allow some horses to perform at an even greater cardiovascular level, increasing their likelihood of winning races and earning more prize money.

### Inositol polyphosphate-5-phosphatase D (*INPP5D*) & SRY-box 5 (*SOX5*)

Also identified by single genome-wide significant SNPs on ECA6 were the *INPP5D* and the *SOX5* genes. The *INPP5D* gene is an important regulator of immune cell signaling, while the *SOX5* gene is involved in embryonic development and has been associated with multiple human diseases and disorders [93–99]. Moreover, both genes have been suggested as important in B cell activity indicating that their association with career earnings in the current study may be rooted in the immune response of a horse [95, 100]. However, mutations in *SOX5* have also been theorized to disrupt neuronal development and function [101, 102].

### Other candidate genes

Regions on ECA1, ECA7, and ECA16 have also previously been described as important for endurance performance traits, while regions on ECA14 and ECA18 associated with gallop racing in other studies do not appear to play a significant role in harness racing [4–8, 10–15, 19–21, 39, 66]. This likely suggest a greater demand for endurance in harness racing compared to gallop racing and is perhaps a sign of the different physiological demands for speed in trot versus speed in gallop. Candidate genes for harness racing success in the current study were also identified on ECA1, ECA2, ECA6, ECA7, ECA16, ECA17, ECA23, ECA25, ECA28, ECA29, and ECA31. However, it is important to note that the MAF threshold applied in the current study is slightly lower than is generally accepted. Although this may have inadvertently resulted in some SNP associations being simply by chance, it is also plausible that the lower MAF threshold allowed for the capture of candidate genes/regions that are perhaps the difference between an elite horse and a very, very good horse. Racing performance is undoubtedly complex and the unique history of the NSCT, being a blend of draught horse and racehorse, means that rare variants cannot be ruled out purely because they are rare – particularly when one considers the rarity of an elite racehorse. While not all candidate variants/genes are discussed above, the results of the current analyses clearly suggest that different molecular and cellular events mediate adaptive processes in the neuromusculoskeletal system in response to exercise. High intensity exercise (e.g. racing) is known to be associated with significant physiological adaptations in the neuromuscular system in equine athletes with prolonged and intense exercise potentially resulting in oxidative damage to cellular constituents [103]. Moreover, the importance of the central nervous system (CNS) as a critical “central governing” factor in sporting performance has been previously documented in endurance horses with exercise shown to induce several biological processes that regulate neurological functions that help to maintain good mental health [104]. Our results add to this line of thought, providing further evidence that genes involved in neural regulations (e.g. *GRIN2B*) likely play an important role in controlling the fundamental biological processes underlying adaptation to equine athletic performance.

## Conclusions

After strict Bonferroni correction, 9 genome-wide significant and 23 suggestive genome-wide significant SNPs associated with harness racing success were identified. These SNPs were located on ECA1, ECA2, ECA6, ECA7, ECA16, ECA17, ECA23, ECA25, ECA28, ECA29, and ECA31 with eight genes (*GRIN2B*, *DOCK8*, *ATP7B*, *KCNRG*, *PIP5K1B, PDE3A, INPP5D, SOX5*) suggested as strong candidate genes for harness racing success. Apart from the physical attributes required to achieve racing success, multiple candidate genes identified in the current study also advocate learning ability and memory as critical to success. However, further analyses of these genes based on additional genetic and functional studies are required to explore this notion in greater detail. Moreover, future studies should also consider a validation study with an independent population as well as sequencing of candidate genes to better identify causal alleles.

## Additional files

Additional file 1: Extent of linkage disequilibrium of the final sample of horses. Pairwise r^2^ was calculated between each SNP within 1 Mb. (TIFF 15316 kb)

Additional file 2: QQ plots for earnings, best km time, and number of gallops analyses. Top panel - corrected QQ plot for earnings analysis (Uncorrected λ = 1.0532); Middle panel – corrected QQ plot for best km time analysis (λ = 1.0902); Bottom panel – corrected QQ plot for number of gallops analysis (λ = 1.0256). (TIF 111 kb)

Additional file 3: Functional classification gene list from PANTHER analysis. (XLSX 27 kb)

## Abbreviations

ATP7B: ATPase copper transporting beta
BT: Best km time
CE: Career earnings
CT: Coldblooded trotter
DMRT3: Doublesex and mab-3 related transcription factor 3
DOCK8: Dedicator of cytokinesis
ECA: *Equus caballus* chromosome
GRIN2B: Glutamate ionotropic receptor NMDA type subunit 2B
GWA: Genome-wide association
INPP5D: Inositol polyphosphate-5-phosphatase D
KCNRG: Potassium channel regulator
NG: Number of gallops
NSCT: Norwegian-Swedish Coldblooded Trotter
PDE3A: Phosphodiesterase 3A
PIP5K1B: Phosphatidylinositol-4-phosphate 5-kinase type 1 beta
QH: Quarter horse
SB: Standardbred trotter
SOX5: SRY-box 5
TB: Thoroughbred
