# Supplementary figures and images for: A genome-wide association study for harness racing success in the Norwegian-Swedish coldblooded trotter reveals genes for learning and energy metabolism
Source: BMC Genet. 2018 Aug 29;19:80. doi: 10.1186/s12863-018-0670-3 (PMC6114527; doi:10.1186/s12863-018-0670-3)

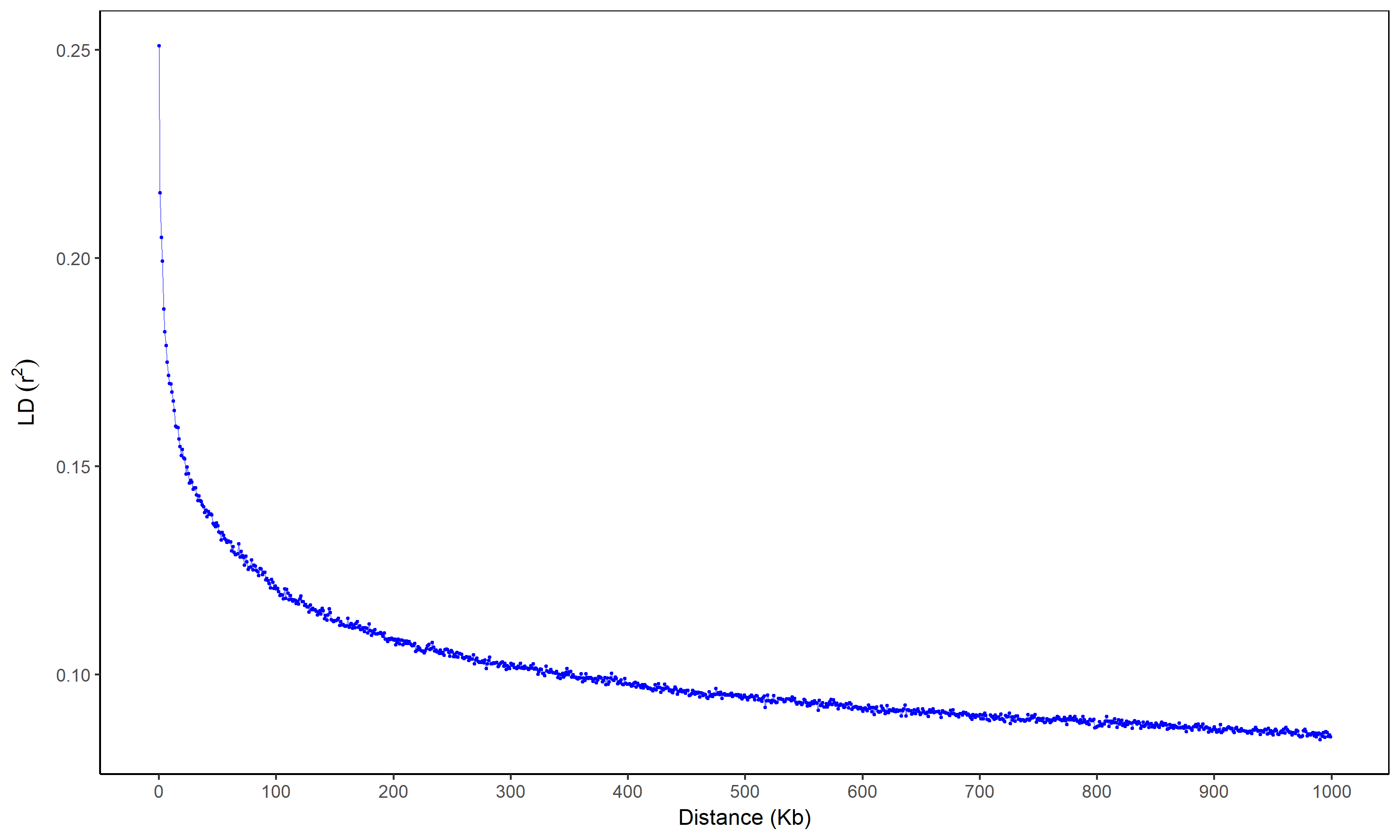

Supplement: Supplementary file 1 — Extent of linkage disequilibrium of the final sample of horses. Pairwise r2 was calculated between each SNP within 1 Mb. (TIFF 15316 kb) [file 12863_2018_670_MOESM1_ESM.tiff]

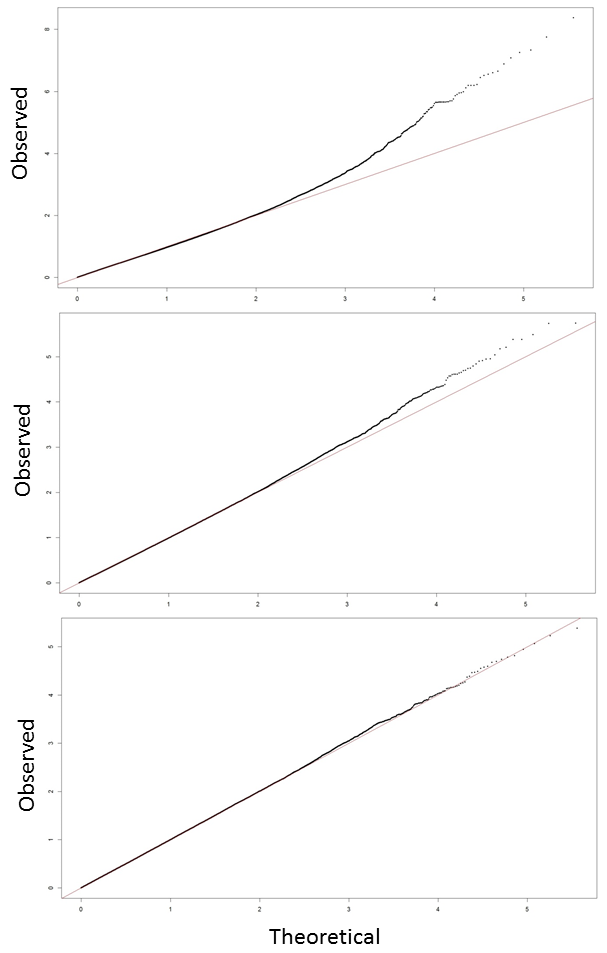

Supplement: Supplementary file 2 — QQ plots for earnings, best km time, and number of gallops analyses. Top panel - corrected QQ plot for earnings analysis (Uncorrected λ = 1.0532); Middle panel – corrected QQ plot for best km time analysis (λ = 1.0902); Bottom panel – corrected QQ plot for number of gallops analysis (λ = 1.0256). (TIF 111 kb) [file 12863_2018_670_MOESM2_ESM.tif]
